# Contributions of nitrogen deposition and forest regrowth to terrestrial carbon uptake

**DOI:** 10.1186/1750-0680-2-5

**Published:** 2007-05-29

**Authors:** Galina Churkina, Kristina Trusilova, Mona Vetter, Frank Dentener

**Affiliations:** 1 Max-Planck Institute for Biogeochemistry, Hans-Knöllstr. 10, 07745 Jena, Germany; 2 European Commission, Institute for Environment and Sustainability, Joint Research Center, Ispra, Italy

## Abstract

**Background:**

The amount of reactive nitrogen deposited on land has doubled globally and become at least five-times higher in Europe, Eastern United States, and South East Asia since 1860 mostly because of increases in fertilizer production and fossil fuel burning. Because vegetation growth in the Northern Hemisphere is typically nitrogen-limited, increased nitrogen deposition could have an attenuating effect on rising atmospheric CO_2 _by stimulating the vegetation productivity and accumulation of carbon in biomass.

**Results:**

This study shows that elevated nitrogen deposition would not significantly enhance land carbon uptake unless we consider its effects on re-growing forests. Our results suggest that nitrogen enriched land ecosystems sequestered 0.62–2.33 PgC in the 1980s and 0.75–2.21 PgC in the 1990s depending on the proportion and age of re-growing forests. During these two decades land ecosystems are estimated to have absorbed 13–41% of carbon emitted by fossil fuel burning.

**Conclusion:**

Although land ecosystems and especially forests with lifted nitrogen limitations have the potential to decelerate the rise of CO_2 _concentrations in the atmosphere, the effect is only significant over a limited period of time. The carbon uptake associated with forest re-growth and amplified by high nitrogen deposition will decrease as soon as the forests reach maturity. Therefore, assessments relying on carbon stored on land from enhanced atmospheric nitrogen deposition to balance fossil fuel emissions may be inaccurate.

## Background

The global climate is expected to change in response to rising concentrations of atmospheric carbon dioxide (CO_2_), because CO_2 _in the atmosphere traps heat. The magnitude of this change depends on the rate of CO_2 _emissions from human activities as well as on carbon uptake by oceans and land. Carbon dioxide fertilization, climate change, nitrogen deposition and land management can enhance carbon uptake on land. A globally significant carbon sink in 1980's-1990's in northern extratropical regions [1] was inferred from variations in atmospheric CO_2 _concentrations. Although this sink was attributed mostly to forest ecosystems [2-5], the magnitude and cause of this sink remain uncertain. Population growth, industrial expansion, and political changes lead to exponentially increasing deposition of reactive nitrogen on land and re-growth of forests, which were identified among the major causes of land carbon sink in the Northern Hemisphere.

Nitrogen is a primary limiting nutrient throughout terrestrial ecosystems of mid and high latitudes, and an important limiting nutrient for plant growth throughout subtropical and tropical ecosystems [6], where phosphorus is a co-limiting or limiting nutrient [7,8]. Additional nitrogen supply through fertilization and atmospheric deposition could reduce or even remove the nitrogen limitation on carbon uptake. Overload of nitrogen deposited on land can lead to decline of ecosystems. Globally the amount of reactive atmospheric nitrogen inputs increased from 41 TgN/yr in 1950 to 103 TgN/yr in 2000 with proportional increase of deposition on land [9]. One third of the global nitrogen inputs entered the land ecosystems and one tenth the forests. Given the high carbon to nitrogen ratios and long lifetimes of carbon in wood, a most significant effect of nitrogen fertilization is expected in forests.

Did land carbon uptake increase in response to the higher nitrogen depositions during the last few decades? Studies addressing this question agreed on the location of major response – the temperate forests located between 25° and 55° north, but disagree on the magnitude of the response. Based on results from a series of ^15^N-tracer field experiments, Nadelhoffer et al. [10] argued that increased inputs of combined nitrogen from atmosphere made a minor contribution to land carbon uptake. Their stoichiometric budget suggested that fertilized temperate forests sequestered only 0.25 PgC per year in addition. In contrast, model based estimates [11,12] showed significant increases in land carbon uptake. Townsend et al. [11] estimated an additional carbon uptake by land in the order of 0.3–1.3 PgC per year, using an ecosystem model and spatially explicit nitrogen depositions from fossil fuels burning (NO_y_). Using the same vegetation model and various predicted spatial distributions of atmospheric nitrogen deposition (including both NO_y _and NH_x_), Holland et al. [12] showed even higher carbon uptake of 1.5–2.0 Pg per year. Forest regrowth was identified as another major driver of elevated carbon uptake in the Northern Hemisphere in 1980–1999. Changing forest management practices lead to increasing fraction of young forests with higher carbon uptake. Depending on the country these young forests were either planted after harvesting or on abandoned agricultural land. Based on forest inventories a carbon sink of 0.11 PgC/yr in Europe and 0.018 PgC/yr in Japan was primarily attributed to regrowth of young forests [2,3]. In China and the United States both forest regrowth and afforestation lead to carbon sink of 0.03 PgC/yr [4] and 0.11–0.15 PgC/yr [5,13] respectively. Russian forests have been reported as a highly variable carbon sink (0.06–0.3 Pg C/yr) during those decades [14]. Although the increased uptake of CO_2 _caused by forest regrowth and afforrestation is relatively undisputed, the relative roles of climate change or land management responsible for this increase has not been defined. In addition, atmospheric nitrogen deposition was exponentially increasing during the same time period. Was nitrogen fertilization leading to a faster forest re-growth and hence to an increased land carbon uptake?

In this study we investigate combined effect of increased nitrogen deposition and forest re-growth on land carbon uptake. We use results of biogeochemical model simulations to show that elevated nitrogen deposition is unlikely to be the major contributor to the increased land carbon sink unless we consider its effects on re-growing forests. The atmospheric nitrogen deposition for 1860–1999 was calculated by the state-of-the-art three dimensional atmospheric chemistry transport model TM3 [15], which provided spatial distributions of reactive atmospheric nitrogen deposition (NO_y _and NH_x_). To assess the effect of enhanced nitrogen deposition on land carbon uptake we used a terrestrial biogeochemical model BIOME-BGC [16-18], which calculates water, carbon, and nitrogen pools dynamics as well as their fluxes. The model considers explicit patterns of nitrogen input and loss from ecosystems (see Methods). To isolate effects of increasing nitrogen deposition we performed model simulations with both increasing CO_2 _and nitrogen deposition as well as with increasing CO_2 _and constant nitrogen deposition. We estimate carbon uptake of land ecosystems assuming that temperate forests were at three different growth stages: 'mature', 'middle-aged', and 'young'.

## Results

**Figure 1:**
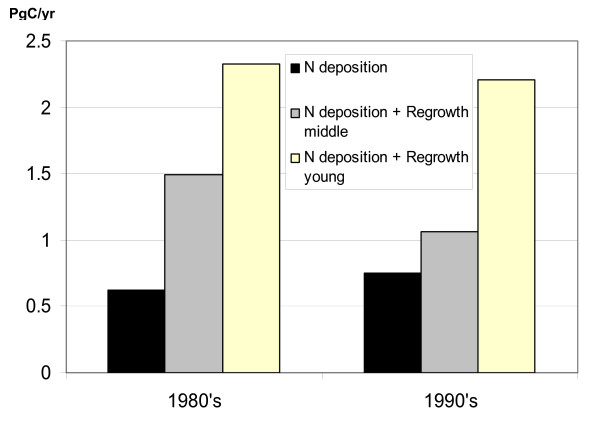
Changes in land carbon uptake in response to increasing nitrogen deposition and both nitrogen deposition and forest regrowth. The presented values are differences in net carbon uptake modelled with and without increasing atmospheric nitrogen deposition for mature, young and middle-aged forests. The bars show global carbon uptake averaged for 1980's and 1990's.

Our results suggest that the net gain of carbon in young forests with lowered nitrogen limitation is higher than in the mature ones. Land vegetation fertilized with reactive nitrogen would take up additional 0.62 Pg C/yr in 1980's and 0.75 Pg C/yr, in the 1990's assuming mature forests in the model simulations (Figure 1). The estimated carbon uptake in both decades was higher in simulations with regrowing forests. The model simulations with forests planted in the 1950 showed that land vegetation would sequester at least 1.5 Pg C/yr (140% more) in the 1980's and 1.06 Pg C/yr (40% more) in the 1990's once nitrogen deposition increased. In the simulation with young forests planted in the 1970 the additional carbon uptake of land would raise up to 2.33 Pg C/yr in the 1980's and 2.21 Pg C/yr in the 1990's or to respectively 270% and 190% of carbon uptake derived in the simulation with mature forests. A result from our model simulations is that young forest grows faster and reaches maturity earlier if amount of nutrients is sufficient to support this growth. In addition young forest generates less litter than mature one and has lower ecosystem respiration. Therefore the effect of increased nitrogen deposition on regrowing forests is considerably higher than on mature forests.

The differences between mature and young forests responses to high nitrogen inputs diminish once re-growing forest matures. Change in land carbon uptake from 1980's to 1990's had opposite trends in simulation with mature and in simulations with re-growing forests. Assuming mature forests our model estimates suggested that in 1990's land absorbed 0.13 Pg of carbon per year more than in 1980's. This increase was associated with rising deposition of reactive nitrogen only (from 80 TgN/yr in 1980's to 95 TgN/yr 1990's). In both simulations with re-growing forests the additional carbon uptake has dropped by 0.24–0.44 PgC/yr from 1980's to 1990's. This drop has occurred because growth of forests was slowing down after the initial stage of fast growth, which was accelerated by higher nitrogen inputs. As the forest ages more biomass is accumulated and ecosystem respiration is increasing. Therefore increased carbon gain in forests can not last forever.

## Discussion

### Land carbon uptake and residual carbon sink

Previous studies [19,20] showed existence of the residual carbon sink, which is an imbalance between annual average emissions of CO_2 _and the sum of the annual carbon accumulation in the atmosphere and the annual carbon uptake by the oceans. This imbalance, attributed to processes on land, has slightly increased from 0.3–4.0 PgC/yr in 1980's to 1.6–4.8 PgC/yr in 1990's. Our results suggest that the effect of nitrogen fertilization on land carbon uptake could explain 20–70% of the residual carbon sink (Table 1) depending on our assumptions of the age, proportion, and distribution of re-growing forests. Our estimates for global additional carbon uptake assuming mature forests are lower than comparable estimates from previous study for unmanaged vegetation [12]. The latter study was based on simulations of a model with an annual time step, which ignored seasonal dynamics of carbon – nitrogen interactions, and used only one nitrogen deposition input averaged for the 1990's. It is possible that these simplified assumption in the model and for the nitrogen inputs lead to overestimation of land carbon response to increasing nitrogen loads. Our results suggest twice higher increase in carbon uptake in temperate forests than a study based on ^15^N tracer field experiments [10], which suggested increase of 0.25 Pg carbon per year for temperate forests. This discrepancy is probably attributable to the small sample size (only nine forests), which was not very representative of temperate forest ecosystems. It could be also related to under-representation of certain ecosystem processes like pathways of plant nitrogen uptake or reactive nitrogen transformations in soil in our modelling approach which is discussed below.

**Table 1:** Residual land carbon sink

**Reference**	1980's (Pg C/yr)	1990's (Pg C/yr)
**Residual land carbon sink**		
IPCC 2001 [19]	-0.3–3.8	incomplete
House et al. [20]	0.3–4.0	1.6–4.8
**Additional land carbon uptake due to increased nitrogen deposition (globally)**		
Townsend et al. [11]	n/a	0.3–1.3
Holland et al. [12]	n/a	1.5–2.0
This study	0.62–2.33	0.75–2.21
**Additional land carbon uptake due to increased nitrogen deposition (temperate forests)**		
Nadelhoffer et al. [10]	n/a	0.25
This study	0.34–1.62	0.4–1.85

Negative values represent atmospheric CO_2_ increase (or land sources), positive numbers depict atmospheric CO_2_ decrease (land sinks).

### Land carbon uptake and fossil fuel emissions

Would this additional carbon accumulated on land offset the fossil fuel emissions? During 1980–1999 approximately 110 Pg of carbon has been emitted into the atmosphere from fossil fuel burning, industry, and deforestation (EDGAR-HYDE 1.4, [21]). Our model predicted that increased atmospheric nitrogen deposition had caused 14, 25, and 45 Pg of additional carbon to accumulate on land during the same time period assuming mature, middle-age, and young forests respectively (compared to a case where we assumed pre-industrial levels of reactive nitrogen deposition). Assuming that age structure of the world forests in 1980–1999 was between ages of the mature and young forests used in model's simulations, increased nitrogen deposition could attenuate rising atmospheric CO_2 _by something between 13% and 41%. The real number lays somewhere in between, most likely closer to the lower limit, since only small fraction of the world forests in 1980's and 1990's were re-growing. Once the forests mature, their ability to take up more carbon will diminish.

### Uncertainties in estimated land carbon uptake

Our budget (Table 1) is subject to some uncertainties related to simplified representation of ecosystem processes as well as land use dynamics in our modelling study. First, our model does not include the mechanism for nitrogen uptake through the stomata of leaves. In closed-canopy forests, forest canopies can intercept atmospheric nitrogen and assimilate retained reactive nitrogen from air. This mechanism was not implemented in the model, because it is not clear how significant the proportion of total incoming inorganic nitrogen intercepted by the canopy is. If this proportion is only 16% as estimated for North American forests [22], then it would not change our results considerably. However if it reaches 40% or more than 90% [23] and all intercepted nitrogen is taken up by foliage then a nitrogen-induced carbon sink may be higher than estimated in our study. Second, our model does not include transformations of reactive nitrogen in the soil, which may be locked up in soil or cause production of dissolved organic nitrogen and carbon. Experiments [24,25] suggest that chronic additions of nitrate to terrestrial ecosystems lead to higher leaching of dissolved organic nitrogen and carbon rather than to plant productivity increase. The mechanisms behind formation of these dissolved organic compounds and conditions under which it occurs still have to be understood. Including this feedback may decrease estimates of land carbon uptake in some regions. Third, we assumed that land cover remained constant during 1980–1999. In reality the land cover has been experiencing changes during the last two decades. The estimates of these changes and their locations however are highly uncertain. Forest area was decreasing globally by approximately 2% per decade [26]. In Europe and North America, forest cover increased by approximately 0.14–0.2% per decade [2,26]. In China forest cover was decreasing by 2.3% per decade according to FAO [26] and increasing by 1.5% per decade according to Fang [4]. Given these uncertainties in forest cover change, we feel that assumption of constant land cover was acceptable in this study.

This study is a frontier research and the results need some confirmation from an independent modelling effort which may include a more detailed treatment of nitrogen cycle and carbon-nitrogen interactions. In addition to the abovementioned processes the next generation of global biogeochemical models may include the following mechanisms: different fates of NHx and NOy within an ecosystem and corresponding effects on ecosystem dynamics; depression of nitrogen mineralization in soils under increasing atmospheric deposition of nitrogen; "N saturation" effects, with N leaching losses approaching N input rates in forests; effect of available nitrogen on carbon allocation within a plant as well as changing plant and soil C:N ratios under chronic N additions. Given complexity of the nitrogen cycle and limitations of any modelling approach, we have to prioritize processes to be included based on their generality and level of understanding.

## Conclusion

In this study we analysed the combined effect of increasing nitrogen deposition and forest re-growth on the global land uptake of carbon. We conclude that elevated nitrogen deposition is unlikely to be the major contributor to the increased land carbon sink unless we consider its effects on re-growing forests. Moreover carbon uptake associated with forest re-growth and amplified by high nitrogen deposition will decrease as soon as forests reach maturity. To refine our estimates of land carbon uptake we would need to know the locations of re-growing forests and their age. We also may need to revise the model's representation of carbon-nitrogen interactions in ecosystems once we better understand different pathways of plant nitrogen uptake, role of nitrogen availability in the allocation of carbon, and formation of dissolved organic nitrogen in soil.

Although fossil fuel burning, intensive agriculture, and forest management increase CO_2 _emissions to the atmosphere, they can also stimulate land carbon uptake, which partially offsets increasing CO2 from fossil fuel emissions. These secondary effects such as increased nitrogen deposition and re-growth of forests should not be considered as purely beneficial. High nitrogen deposition can lead also to nitrogen saturation which, depending on ecosystem type, can cause forest decline [27,28] or dramatic increases in export of dissolved organic carbon and nitrogen from forest ecosystems to streams, rivers, lakes, and coastal systems [24]. High NO_x _emissions lead to ozone production [29], which can have damaging effects on plant growth.

## Methods

### Model description

To assess the effect of enhanced nitrogen deposition and CO_2 _on European carbon uptake we used a terrestrial biogeochemical model BIOME-BGC (version 4.1.1 with carbon and nitrogen allocation routine from 4.1), which calculates water, carbon, and nitrogen pools dynamics as well as their fluxes on a daily basis[16,30]. The model is driven by maximum and minimum air temperatures, precipitation, air humidity, and solar radiation data. Carbon dynamics include calculations of the plant growth onset and senescence periods, allocation of assimilates to the different plant organs, mortality as well as litter production, and soil organic matter decomposition. Nitrogen dynamics include calculations of plant and soil microbial demands based on carbon to nitrogen ratios of plant organs, litter, and soil microbial community. The amount of nitrogen available to satisfy these demands is determined by nitrogen deposited from atmosphere, biological nitrogen fixation and nitrogen mineralized during soil organic matter decomposition. Nitrogen loss from ecosystem is determined by the amount of soluble mineral nitrogen available, water outflow, and soil water content.

Possible forest decline caused by high nitrogen inputs were not included in the model, because it was not relevant on the coarse grid scale considered in this study.

The Biome-BGC model was successfully evaluated for a number of hydrological and carbon cycle components [31-33]. In recent years, the model has been corroborated with eddy-covariance data at the sites with high and low deposition of atmospheric nitrogen [17,18,34,35].

### Model parameterization

The model was parameterized for seven vegetation types: deciduous broadleaf forest, evergreen needleaf forest, evergreen broadleaf forest, evergreen deciduous forest, scrubland, C4 and C3 grasslands. Ecophysiological parameters (e.g. carbon to nitrogen ratios of forest ecosystem's pools) for evergreen needleaf and deciduous broadleaf forests were optimized from field measurements of net carbon fluxes [36]. Parameterizations for evergreen broadleaf forest, deciduous needleaf forest, grasslands, and shrubland were as in White et al. [37].

### Model input data and simulations

All input data were transformed to 1° × 1° spatial resolution and subsequent model simulations were performed at this spatial resolution as well. Input land surface characteristics included digital elevation map, soil texture map, and land cover classification. Since in this study we did not consider land use changes, for all spatially explicit simulations of carbon, nitrogen, and water fluxes, a vegetation map [38] was held constant.

The model was first run with constant annual atmospheric nitrogen deposition (2 kgN/ha yr, Holland, 1999 #728) and CO_2 _concentrations (283 ppm) as well as daily climate data from NCEP Reanalysis [39] for 1948–1957 at a spatial resolution of 1° × 1° until an ecological equilibrium was reached.

#### Simulations with mature and re-growing forests

**Figure 2:**
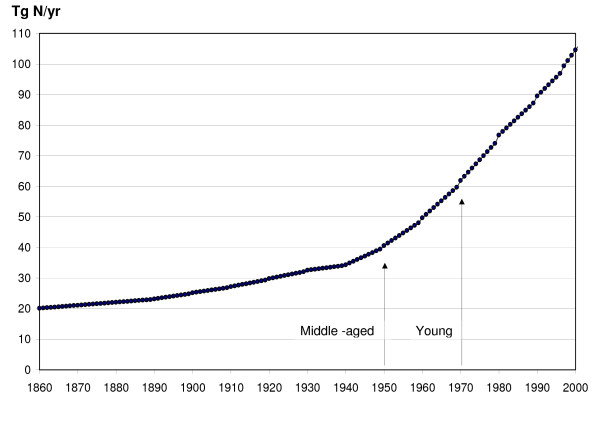
Changes in annual atmospheric nitrogen deposition based on TM3 model simulations from 1860 to 2000. Black arrows on the plot show years when forests were assumed to be planted in simulations with middle-aged and young forests.

Previous studies [40] suggested age-related response of forest growth to changing abiotic conditions (increased temperature, CO_2 _and nitrogen deposition). To capture these effects we estimated carbon uptake of three different groups of forests: mature forests planted long before nitrogen deposition started to increase; middle age forests planted when the nitrogen deposition was still relatively low (1950), but exponentially increasing thereafter; and young forests planted when nitrogen deposition was already high (1970) and exponentially increasing during their lifetime (Figure 2).

We isolated the effects of elevated nitrogen deposition on forests at different succession stages by simulations with CO_2 _increase, but no nitrogen deposition increase and with both CO_2 _and nitrogen increases. In each simulation we assumed that forests had the same age distribution.

#### Simulation with increasing CO_2_

Between 1860 and 1999, ambient CO_2 _increased from 283 ppm to 368 ppm. To isolate the effect of increased CO_2 _on carbon uptake we performed model simulations with nitrogen deposition kept constant at 1860 level and CO_2 _changing as described above.

#### Simulation with increasing CO^_2_^ and nitrogen deposition

To isolate the additional effect of increased nitrogen deposition on carbon uptake we performed model simulations with changing both atmospheric CO_2 _and nitrogen deposition.

The BIOME-BGC model simulations were driven by the state-of-the-art atmospheric nitrogen deposition for 1860–1999 (Figure 2). Spatial distribution of atmospheric nitrogen deposition was estimated with three dimensional atmospheric chemical transport model TM3 [15]. The estimates included deposition of both NO_y _and NH_x_, which were added to get the total atmospheric nitrogen deposition.

These data were produced using the TM3 global chemistry transport model that has a horizontal resolution of 5° longitude and 3.75° latitude, and has 19 vertical levels to 10 hPa. It is driven by six-hourly meteorological data obtained from the European Centre for Medium-Range Weather Forecasting. Mid-1990s deposition rates are based on Rodhe et al. [15] combining output for reduced and oxidized nitrogen deposition (both wet and dry). Comparisons between modelled and measured deposition rates show model accuracy to be within 50% (and often substantially better). The main sources of error arise from emissions inventories, atmospheric transport, removal, and chemical transformations. The original annual model outputs at decadal time step were transformed to annual outputs at one year time step using linear interpolation for each grid pixel.

## Competing interests

The author(s) declare that they have no competing interests.

## Authors' contributions

GC performed analysis of the model simulations and wrote the manuscript. KT and MV performed simulations with BIOME-BGC model. FD provided outputs from atmospheric chemical transport model TM3. All authors read and approved the final manuscript.

## References

[B1] Schimel DS, House JI, Hibbard KA, Bousquet P, Ciais P, Peylin P, Braswell BH, Apps MJ, Baker D, Bondeau A, Canadell JG, Churkina G, Cramer W, Denning AS, Field CB, Fridlingstein P, Goodale C, Heimann M, Houghton RA, Melillo JM, Moore III B, Murdiyarso D, Noble I, Pacala SW, Prentice IC, Rauparch MR, Rayner PJ, Scholes RJ, Steffen WL, Wirth C (2001). Recent patterns and mechanisms of carbon exchange by terrestrial ecosystems. Nature.

[B2] Nabuurs GJ, Schelhaas MJ, Mohren GMJ, Field CB (2003). Temporal evolution of the European forest carbon sink from 1950 to 1999. Global Change Biology.

[B3] Fang J, Oikawa T, Kato T, Mo W, Wang Z (2005). Biomass carbon accumulation by Japan's forests from 1947 to 1995. Global Biogeochemical Cycles.

[B4] Fang J, Chen A, Peng C, Zhao S, Ci L (2001). Changes in forest biomass carbon storage in China between 1949 and 1998. Science.

[B5] Caspersen JP, Pacala SW, Jenkins JC, Hurtt GC, Moorcroft PR, Birdsey RA (2000). Contribution of land-use history to carbon accumulation in U.S. forests. Science.

[B6] Vitousek P, Edin LO, Matson PA, Fownes JH, Neff J, Pace M and Groffman P (1998). Within-system element cycles, input-output budgets, and nutrient limitations. Success, Limitations, and Frontiers in Ecosystem Science.

[B7] Tanner EVJ, Vitousek P, Cuevas E (1998). Experimental investigation of nutrient limitation of forest growth on wet tropical mountains. Ecology.

[B8] D'Antonio C, Mack MC (2006). Nutrient limitation in a fire-derived, nitrogen rich Hawaiian grassland. Biotropica.

[B9] Galloway JN, Dentener FJ, Capone DG, Boyer EW, Howarth R, Seitzinger SP, Asner G, Cleveland CC, Green PA, Holland EA, Karl DM, Michaels AF, Porter JH, Townshend AR, Vorosmarty CJ (2004). Nitrogen cycles: past, present, and future. Biogeochemistry.

[B10] Nadelhoffer KL, Emmett BA, Gundersen P, Kjønaas OJ, Koopmans CJ, Schleppi P, Tietema A, Wright R (1999). Nitrogen deposition makes a minor contribution to carbon sequestration in temperate forests. Nature.

[B11] Townsend AR, Braswell BH, Holland EA, Penner JE (1996). Spatial and temporal patterns in terrestrial carbon storage due to deposition of fossil fuel nitrogen. Ecological Applications.

[B12] Holland EA, Braswell BH, Lamarque JF, Townsend A, Sulzman J, Müller JF, Dentener F, Brasseur G, Levy II H, Penner JE, Roelofs GJ (1997). Variation in the predicted spatial distribution of atmospheric nitrogen deposition and their impact on  carbon uptake by terrestrial ecosystems. Journal of Geophysical Research.

[B13] Pacala SW, Hurtt GG, Baker D, Peylin P, Houghton RA, Birdsey RA, Heath L, Sundquist ET, Stallard RF, Ciais P, Moorcroft P, Caspersen JP, Shevliakova E, Moore B, Kohlmaier G, Holland EA, Gloor M, Harmon ME, Fan S, Sarmiento J, Goodale C, Schimel DS, Field CB (2001). Consistant land- and atmosphere-based U.S. carbon sink estimates. Science.

[B14] Shvidenko A, Nilsson S (2002). Dynamics of Russian forests and the carbon budget in 1961-1998: an assesment based on long-term forest inventory data. Climatic Change.

[B15] Rodhe H, Dentener FJ, Schulz M (2002). The global distribution of acidifying wet deposition. Environmental Science and Technology.

[B16] Running SW, Hunt ERJ, Ehleringer JR and Field CB (1993). Generalization of a forest ecosystem process model for other biomes, Biome-BGC, and an application for global-scale models. Scaling Physiological Processes: Leaf to Globe.

[B17] Thornton PE, Law BE, Gholz HL, Clark KL, Falge E, Ellsworth DE, Goldstein AH, Monson RH, Hollinger DY, Falk M, Falk JP (2002). Modeling and measuring the effects of disturbance history and climate on carbon and water budgets in evergreen needleleaf forests. Agricultural and Forest Meteorology.

[B18] Churkina G, Tenhunen J, Thornton PE, Elbers JA, Erhard M, Falge E, Grünwald T, Kowalski AS, Rannik, Sprinz DF (2003). Analyzing the ecosystem carbon dynamics of four European coniferous forest using a biogeochemistry model. ECOSYSTEMS.

[B19] Prentice IC, Farquhar GD, Fashm M, Goulden ML, Heimann M, Jaramillo V, Kheshgi H, Le Quéré C, Scholes RJ, Houghton RA, Ding Y, Griggs DJ, Noguer M, van der Linden J, Dai X, Maskell K and Johnson CM (2001). The carbon cycle and atmospheric carbon dioxide. Climate Change 2001: The scientific basis.

[B20] House JI, Prentice IC, Ramankutty N, Houghton RA, Heimann M (2003). Reconciling apparent inconsistencies in estimates of terrestrial CO2 sources and sinks. Tellus.

[B21] Van Aardenne JA, Dentener FJ, Olivier JGJ, Klein Goldewijk CGM, Lelieveld J (2001). A 1 x 1 degree resolution dataset of historical anthropogenic trace gas emissions for the period 1890-1990. Global Biogeochemical Cycles.

[B22] Johnson DW, Lindberg SE (1992). Atmospheric Deposition and Forest Nutrient Cycling. Ecological Studies, Vol 91.

[B23] McLaughlin JW, Fernandez IJ, Richards KJ (1996). Atmospheric deposition to a low-elevation spruce-fir forest, Main, USA. Journal of Environmental Quality.

[B24] Pregitzer KS, Zak DR, Burton AJ, Ashby JA, Macdonald NW (2004). Chronic nitrate additions dramatically increase the export of carbon and nitrogen from northern hardwood ecosystems. Biogeochemistry.

[B25] Currie WS (1996). Vertical transport of dissolved organic C and N under long-term N ammendments in pine and hardwood forests. Biogeochemistry.

[B26] FAO FAOSTAT: Land Use.

[B27] Moffat AS (1998). Global nitrogen overload problem grows critical. Science.

[B28] Schulze ED (1989). Air pollution and forest decline in a spruce (Picea abies) forest. Science.

[B29] Chameides WL, Kasibhatla PS, Yienger J, Levy II H (1994). Growth of continental scale metro-agro-plexes, regional ozone pollution, and world food production. Science.

[B30] Thornton PE (1998). Regional Ecosystem Simulation: Combining Surface- and Satellite-Based Observations to Study Linkages between Terrestrial Energy and Mass Budgets. School of Forestry.

[B31] Band LE, Peterson DL, Nemani RR, Running SW (1993). Forest ecosystem processes at the watershed scale: Incorporating hillslope hydrology. Agricultural and Forest Meteorology.

[B32] Running SW (1994). Testing FOREST-BGC ecosystem process simulations across a climatic gradient in Oregon. Ecological Applications.

[B33] Churkina G, Running SW (2000). Investigating the balance between timber harvest and productivity of the global coniferous forests under global change. Climatic Change.

[B34] Cienciala E, Running SW, Lindroth A, Grelle A, Ryan MG (1998). Analysis of carbon and water fluxes from the NOPEX boreal forest: comparison of measurments with Forest-BGC simulations. Journal of Hydrology.

[B35] Law BE, Sun OJ, Campbell J, van Tuyl S, Thornton PE (2003). Changes in carbon storage and fluxes in chronosequence of ponderosa pine. Global Change Biology.

[B36] Trusilova K, Churkina G, Vetter M, Reichstein M, Schumacher J, Knohl A, Rannik, Grünwald T, Moors E, Granier A Parameter estimation for the terrestrial ecosystem model BIOME-BGC using nonlinear inversion. Ecological Modelling.

[B37] White MA, Thornton PE, Running SW, Nemani RR (2000). Parameterization and sensitivity analysis of the BIOME-BGC terrestrial ecosystem model: Net primary production controls. Earth Interactions.

[B38] DeFries R, Hansen M, Townshend J, Sohlberg R (1998). Global land cover classification at 8 km spatial resolution: The use of training data derived from Landsat imagery in decision tree classifiers. International Journal of Remote Sensing.

[B39] Kalnay E, Kanamitsu M, R. Kistler WC, S. Saha GW, J. Janowiak KCM, Joseph D (1996). The NCEP/NCAR reanalysis project. Bulletin of American Meteorologocal Society.

[B40] Vetter M, Wirth C, Böttcher H, Churkina G, Schulze ED, Wutzler T, Weber G (2005). Partitioning direct and indirect human-induced effects on carbon sequestration of managed coniferous forests using model simulations and forest inventories. Global Change Biology.

